# Hypoglycemia as a Symptom of Neoplastic Disease, with a focus on Insulin-like Growth Factors Producing Tumors

**DOI:** 10.7150/jca.30472

**Published:** 2019-10-20

**Authors:** Jan Schovanek, Lubica Cibickova, Filip Ctvrtlik, Zbynek Tudos, David Karasek, Maurizio Iacobone, Zdenek Frysak

**Affiliations:** 1 Department of Internal Medicine III - Nephrology, Rheumatology and Endocrinology, Faculty of Medicine and Dentistry, Palacky University Olomouc and University Hospital Olomouc, Czech Republic.; 2 Department of Radiology, Faculty of Medicine and Dentistry, Palacky University Olomouc and University Hospital Olomouc, Czech Republic.; 3 Endocrine Surgery Unit, Department of Surgery, Oncology and Gastroenterology, University of Padova, Italy, Via Giustiniani 2, 35128 Padova, Italy.

**Keywords:** Nonislet cell tumor (NICT), Nonislet cell tumor hypoglycemia (NICTH), insulin-like growth factor 2 (IGF-II), Adrenal Glands

## Abstract

This article reviews the current knowledge of uncommon causes of hypoglycemia, with a focus on neoplastic disease. However, these situations are rare. They commonly accompany severely ill patients and therefore a proper diagnosis is the basis for relevant treatment. Here we discuss the pathophysiological foundation of hypoglycemia - situations caused by increased insulin production or sensitivity - but we also focus on different cytokines which could cause hypoglycemia, especially IGF-II production in what are called nonislet cell tumors. From the clinical perspective we can divide the patients who are affected into "seemingly ill” or “healthy patients” and lead the diagnostic process accordingly.

## Introduction

A drop in glucose serum levels leading to hypoglycemia becomes fully manifested by neuroglycopenic symptoms with changes in level of consciousness. Attending physicians face difficult situations resulting from a plethora of potential causes, clinically presenting as the classic Whipple's triad of a low blood glucose level, clinical symptoms of hypoglycemia at the time of the low glucose level, and the relief of those symptoms after the restoration of glucose levels. The symptoms of hypoglycemia depend on the drop in the glucose level and can be categorized as *neurogenic* or *neuroglycopenic* 1. Neurogenic symptoms are connected to sympathoadrenal activation and include sweating, shakiness, tachycardia, anxiety, and a sensation of hunger. Neuroglycopenic symptoms are caused by the affection of neuronal functions and include weakness, tiredness, and, in extreme cases, coma or eventually death.

In an outpatient setting, apart from the inadequate dosing of glucose-lowering drugs (whether intentional or unintentional), we must think of diet error and alcohol abuse. In a hospital setting, in addition to the above-mentioned causes, we also have to think of errors in the administration of medication. Hospitalized patients also tend to be more ill than patients in outpatient clinics. The patients at higher risk of hypoglycemia are those suffering from malnutrition, severe sepsis, or liver, kidney, or heart failure. On the endocrine ward we can face hypoglycemia as a sign of endocrine disruption caused by hypopituitarism, hypocorticism, or low glucagon levels. On the surgical ward, hypoglycemia could develop as a result of gastric bypass surgery, dumping syndrome, and other post-surgical conditions.

Hypoglycemia accompanying neoplastic diseases, known as tumor-related hypoglycemia, is a separate topic and can, on the basis of its pathogenesis, be divided into four groups; see Table 1. Hypoglycemia with high insulin levels presenting a symptom of a neuroendocrine tumor - insulinoma (islet cell tumor) or functional disorder of pancreatic β-cells - nesidioblastosis. However, in many of the cases of what is called tumor-induced hypoglycemia the etiological agent of hypoglycemia is not an insulin but its structurally similar polypeptide, somatomedin A also called insulin-like growth factor II (IGF-II), and the hypoglycemia is referred to as nonislet cell tumor hypoglycemia (NICTH) 2. Other substances produced by the tumor could also interfere with glucose metabolism, including insulin receptor antibodies and various cytokines (tumor necrosis factor-α, interleukin-1 and -6) and catecholamines (in pheochromocytomas). Finally, hypoglycemia could be directly related to the growth of the tumor - destruction of the liver or adrenal gland by infiltration of the tumor 3. In all cases we should think about the patient in a complex way and the potential causes of hypoglycemia, not omitting the rare causes, as they are the focus of this review.

### Pathophysiology

Humans have very effective systems for the prevention of hypoglycemia 4 and the value of the plasma glucose level itself is not sufficient to show the full image of this clinical condition. Even healthy people exhibit clinical symptoms of hypoglycemia if their glucose level drops below 3 mmol/l 5.

Insulin-like Growth Factors I and II (IGF-I, IGF-II) have well-described structural similarities to insulin and together form a group of what are called somatomedins 6. Hormones fulfill their functions by acting on several types of signaling tyrosine kinase receptors, mainly the insulin receptor (IR) and insulin-like growth factor I receptor (IGF-IR). IGF-II also binds to the non-signaling IGF type 2 receptor (IGF-IIR), which regulates the amount of circulating and tissue IGF-II by transporting the ligand into the cell and degrading it 7. The glucose-lowering effect of IGFs is 10 times lower than that of insulin, but in healthy subjects the serum concentration of IGFs is about 1000 times higher than that of insulin 8. However, in contrast to insulin, in circulation most of the IGFs (90%) are tightly bound to IGF-binding proteins (IGFBPs) 2. IGF-I suppresses liver glucose production, stimulates peripheral glucose utilization, and has an inhibitory effect on insulin secretion. Its role is important for childhood growth and during adulthood it has an anabolic effect. The synthesis of IGF-I largely depends on its stimulation by growth hormone (GH) through the GH receptor, whereas the synthesis of IGF-II is relatively independent of GH action 2. IGF-II is normally produced by the liver and plays an important role in fetal and postnatal development; during this period an A-isoform of an insulin receptor is the main receptor for IGF-II. This isoform of IR is predominantly expressed during prenatal life and enhances the effects of IGF-II during embryogenesis and fetal development 9. Not much is known about the physiological functions of IGF-II in adulthood. Most of the IGF-II produced by the liver forms stabilizing tertiary complexes with a molecular weight of 150-kD with IGF-binding protein 3 (IGFBP3). These large complexes do not interact with the insulin receptor and therefore do not cause hypoglycemia 10. On the contrary, in a patient suffering from what are called nonislet cell tumors the IGF-II can be produced as a result of abnormal post-translational processing in the form of a “big” molecule with a molecular weight of 11-18kD. It seems likely that in many neoplastic cells the levels of the various enzymes involved in post-translational processing are not sufficient to handle the relatively high amounts of “big” IGF-II that are produced adequately 2. This later binds to the IGF-binding protein 2 (IGFBP2), forming smaller binary complexes with a molecular weight of 50-kD (in contrast to physiological tertiary complexes with IGFBP3) and also a fraction of the IGF-II stays in the free unbound form, as reported by Frystyk et al., and the free form of IGF-II was found to be increased 20-fold (not discriminating between free mature and free “big” IGF-II) in comparison to control subjects; however, the levels of total IGF-II were normal 11 or could even be decreased 12. Altogether, in the serums of patients suffering from NICTH increased levels of free IGF-II were found, and also of “big” IGF-II with greater capillary permeability, and thus increased IGF bioavailability to the tissues; these combinations can lead to hypoglycemia as a result of increased glucose utilization in the skeletal muscles and also suppressed gluconeogenesis and glycogenolysis in the liver 10, 11. Moreover, because of increased negative feedback on growth hormone (GH) production by the anterior pituitary gland, the synthesis of GH and GH-dependent IGFBP-3 is reduced - closing the hypoglycemic loop.

Even though the data are not always consistent, it is nowadays believed that IGFs in general and IGF-II in particular can promote tumor growth *in situ* in an autocrine or paracrine fashion once the tumor has been established. Increased expression of IGF-I, IGF-II, and IGF-IR has been determined in a variety of neoplasia, including brain tumors, mammary carcinoma, gastrointestinal cancer, including pancreatic carcinoma, and ovarian carcinoma 13, 14.

### Clinical perspective

The sensation of hypoglycemia can differ broadly between people, as clinicians experienced in performing *insulin tolerance tests* are aware. The latest *Endocrine Society Clinical Practice Guideline* recommends as an initial step in the diagnostic approach to a patient presenting with hypoglycemia without evidence of diabetes mellitus treatment to review the patient's history, physical findings, and all available laboratory data in order to seek clues pointing to specific disorders - drugs, critical illness, hormone deficiencies, and also NICTH, or, in patients with no obvious cause of hypoglycemia, “seemingly well people”, to measure plasma glucose, insulin, C-peptide, proinsulin, and β-hydroxybutyrate (representative of ketones) and to screen for oral hypoglycemic agents (ideally all available sulfonylureas and glinides) and then correct the hypoglycemia with the injection of 1.0 mg glucagon iv with measurement of the plasma glucose response. These data will distinguish endogenous (and exogenous) hyperinsulinemia from other causes of hypoglycemia 4. Additionally, the measurement of insulin antibodies is recommended, however, not necessarily during the hypoglycemia. Their presence indicates rare insulin autoimmune hypoglycemia 15.

In “seemingly well people” endogenous hyperinsulinemia must be evaluated by the above-mentioned laboratory tests and then confirmed by imaging studies and in indicated cases by biopsy. Insulinoma as a solid source of insulin overproduction can be imaged with low sensitivity and specificity by abdominal ultrasound; better clinical usability is obtained with computed tomography, nuclear magnetic resonance, and scintigraphy imaging. The gold standard to confirm insulinoma is to perform endoscopic ultrasonography of the pancreas, which also allows for a biopsy; however, although this procedure is highly valued by clinicians, its performance is highly expert-dependent and thus available only in specialized centers 16.

If insulinoma is excluded, another possible cause of endogenous insulin overproduction is nesidioblastosis - hypertrophy/hyperplasia of pancreatic β-cells 17. Its confirmation is extremely complicated and demanding. Thompson et al. used with high specificity and sensitivity selective arterial calcium stimulation with hepatic venous sampling in 116 patients 18. The correct interpretation of the results that are obtained is conditioned by good knowledge of the pancreatic arteries. Surgery could be a curative option for these patients 18-20.

Pheochromocytoma (PHEO) as a tumor of the adrenal gland has a complex effect on glucose homeostasis. Twenty-five to seventy-five percent of patients with PHEO have altered glucose tolerance 21, 22. Catecholamines (especially norepinephrine) stimulate α2 adreno-receptors and thus inhibit insulin secretion and also increase insulin resistance 22. On the other hand, hypoglycemia was also reported in patients with PHEO, most commonly in the postoperative phase, probably as a result of the sudden loss of catecholamines 23. In this case hypoglycemia manifests with classic symptoms, which could, however, be masked by the effect of residual anesthesia and the presence of β-blockage 24. Severely sick patients with PHEO can present with hypoglycemia as a result of liver metastasis (low glycogen stores) or secondarily as a result of direct consumption of glucose by the tumor (case report of a large tumor of the adrenal gland) 25. In some patients the domination of β2 adreno-receptors effect of catecholamines (esp. epinephrine) results in insulin release and consequent hypoglycemia, usually in conditions where glycogen stores were depleted 26.

Nonislet cell tumor hypoglycemia, also called *Doege-Potter syndrome*, is a rare disease with an estimated incidence of one case per million 27, 28. NICTH, as excellently reviewed by *de Groot et al.*, is thought to be rather a fasting hypoglycemia characterized by diminished hepatic glucose production resulting from the inhibition of glycogenolysis and gluconeogenesis; diminished lipolysis in adipose tissue resulting in low levels of serum-free fatty acids and increased peripheral glucose consumption 2. These symptoms instigating towards insulin-like activity are, however, caused by tumor production of insulin-like growth factor. Those clinical signs and symptoms were described in patients with mesenchymal tumors, fibromas, carcinoid, myelomas, lymphomas and hepatocellular, and colorectal carcinomas 28.

A diagnosis of NICTH is based upon clinical and the above-mentioned laboratory evaluation; in seemingly ill patients the diagnosis might be more straightforward than in healthy-looking people in whom hypoglycemia would be the first symptom of the underlying cancer 29. The measurement of total, free, or “big” IGF-II is not routinely available and is also unnecessary for establishing the diagnosis. Chromatography is considered as a gold standard for IGF-II measurement; however, the total levels of IGF-II might not be significantly changed, as explained above; rather, the ratio of produced “big” IGF-II/IGF-II might be of clinical importance, as might the ratio of IGF-I/IGF-II 29, 30. There is no unifying statement as to whether the existence of NICTH is of prognostic value with respect to predicting the degree of malignancy of a tumor or the (disease-free) survival of the patient 2, 31.

**Figure 1:**
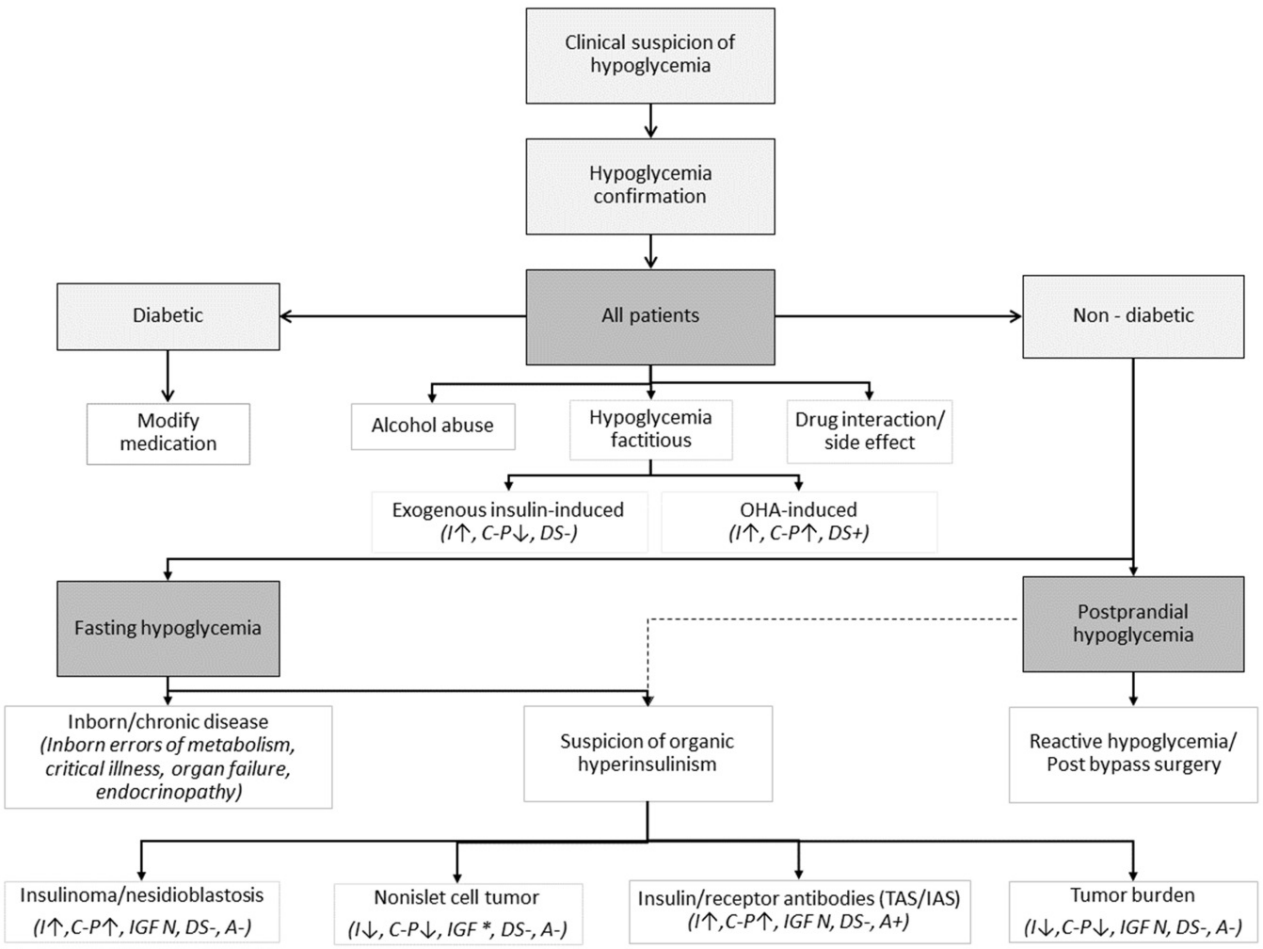
Clinical guidance on potential causes of hypoglycemia with some corresponding laboratory findings in cases of tumor-related hypoglycemia. Modified from 3, 4, 32, 37, 38. Shortcuts: OHA - oral hypoglycemic agents, I - insulin, C-P - C-peptide, DS - drug screen, IGF * - increased pro-IGF-II, free IGF-II, IGF-II/IGF-I ratio, IAS - Insulin Autoimmune Syndrome (“Hirata disease”), TAS - Tumor Autoimmune Syndrome, A - insulin antibody (presence of either anti-insulin antibodies [insulin autoimmune syndrome, Hirata disease] or anti-insulin receptor antibodies [type B insulin resistance]. If TAN antibodies are produced by the tumor, ↑ - increased, ↓ - decreased, + - present, - - absent, N - normal

The diagnostic approach could be guided by Figure 1.

### Treatment

Like any hypoglycemic state, NICTH requires proper treatment by glucose or glucagon. The mainstay of the NICTH is treatment of the primary tumor; complete removal of the tumor mass is curative and leads to the restitution of normoglycemia, but for many patients it is often delayed or unfeasible. There is no clear “standard of care” for managing those tumors 32. In selected cases systemic or local chemotherapy, tumor embolization, or radiation therapy might be effective; however, the overall experience with these methods tends rather not to support their use 32. A common first-line measure is to increase the oral intake of nutrition, sometimes accompanied by parenteral nutrition; however, while this could help to stabilize glucose levels, it cannot serve as a permanent solution. The most effective treatment, and also the one with the most consistent results, seems to be glucocorticoid therapy. It has an immediate effect on the hypoglycemia and also corrects the metabolism in the long term or before curative surgery can be performed.

Glucocorticoids stimulate gluconeogenesis, and at least partially suppress “big” IGF-II and correct biochemical abnormalities involving the GH-IGF axis 2, 5, 33. The use of recombinant human growth hormone (rhGH) has been successful in many cases, as it suppresses peripheral glucose intake and increases levels of IGF-I and IGF-II binding proteins. Combination therapy with glucocorticoids and rhGH might potentiate the benefits of those medications and at the same time prevent some side-effects 34; however, some do not recommend its use except in cases of palliative treatment 31.

The first-line and only curative option for insulinoma is surgical excision by enucleation or partial pancreatectomy 3. In cases of irresectable or malignant tumors (about 5-10% of cases) medical therapy with diazoxide or somatostatin analogs could be used to control hypoglycemic symptoms. However, the data on the use of somatostatin analogs are very inconsistent; their use in extremely resistant cases might be advocated. Experience with systemic chemotherapy in malignant insulinoma is limited. The traditional regimen of choice has been streptozocin and doxorubicin, but uncertainty as to its efficacy, as well as the toxicity of this regimen (nausea, prolonged myelosuppression, renal failure), has prevented its widespread acceptance. Molecularly targeted therapy options with small molecule tyrosine kinase inhibitors and inhibitors of the mammalian (mechanistic) target of rapamycin (mTOR), as well as peptide receptor radioligand therapy, are expected 35.

In pheochromocytoma, hypoglycemia is mainly caused by the effect of insulin; therefore the standard hypoglycemia treatment options should be effective 36. The same is valid for the most common case of postoperative hypoglycemia, which needs to be correctly diagnosed in order to be promptly managed with glucose intravenous therapy 24.

## Conclusions

Hypoglycemia is a common clinical condition. Hypoglycemia in severely ill or oncology patients is not rare; however, the establishment of a correct diagnosis of NICTH requires a good level of knowledge and especially the courage to think about a rare diagnosis. When we are thinking about NICTH, the diagnosis itself is not so difficult to establish.

## Figures and Tables

**Table 1:** Pathophysiological classification of tumor-related hypoglycemia. Modified from 3.

Islet Cell Tumors	Eutopic insulin production
	Insulinoma
**Nonislet Cell Tumors**	Neuroendocrine tumors
	Mesenchymal tumors
	Adrenal, Hepatocellular, Gastrointestinal, Ovarian carcinomas
	Ectopic insulin production (Pheochromocytoma, Small-cell carcinomas)
**Massive tumor burden/liver infiltration**	
**Tumor production of autoantibodies against insulin or the insulin receptor**	
